# What Can We Learn from Sex Differences in MS?

**DOI:** 10.3390/jpm11101006

**Published:** 2021-10-07

**Authors:** Patricia K. Coyle

**Affiliations:** Department of Neurology, Stony Brook University, Stony Brook, NY 11794, USA; patricia.coyle@stonybrookmedicine.edu

**Keywords:** multiple sclerosis, sex differences, gender issues, pregnancy, reproductive counseling

## Abstract

Multiple sclerosis (MS) is the major acquired central nervous system disease of young adults. It is a female predominant disease. Multiple aspects of MS are influenced by sex-based differences. This has become an important area of research and study. It teaches us how the impact of sex on a disease can lead to new insights, guidelines, management, and treatments.

## 1. Introduction

Multiple sclerosis (MS) is a major neurologic disease of unclear etiology and without a cure. It is permeated by sex-based differences. Sex influences multiple aspects of MS, including incidence, expression, activity, prognosis, comorbidities, and outcomes. It is believed that understanding sex-based differences will provide important insights into MS pathophysiology and treatment.

This article will start with a brief review of MS, consider male-related issues, and then spend the rest of the review on female-focused disease aspects. The summary will highlight key sex-based insights.

### 1.1. Multiple Sclerosis

MS is the major acquired central nervous system (CNS) disease of young adults. It involves the brain, spinal cord, and optic nerves. It affects close to a million Americans, and 2.8 million individuals worldwide [1]. MS has a number of interesting and rather unique features for a major neurologic disease. It is female predominant and highly variable. Geographic distribution is uneven, with documented low, medium, and high-risk zones. MS largely affects white ethnicities of Northern European/Scandinavian background, although it is often seen in black populations, and in people from Asia, Latin America, and from across Africa. It affects young people; roughly 90% of MS individuals present between the ages of 15 and 50 years. MS is unusual at the age extremes; 10% or less present after age 50 years, and 1% after age 60 years. Pediatric MS, presenting before age 18, makes up only 2% to 5% of all MS, and 1% or less will present under age 11 years. MS numbers begin to rise following puberty.

MS likely exists for some years before it clinically presents. In fact, in appreciating the spectrum of this disease, autopsy studies have estimated that up to 25% of individuals who have the pathologic disease may never show clinical expression (Table 1) [2]. Such patients can be detected as having a radiologically isolated syndrome (RIS) if they happen to have a coincidental CNS magnetic resonance imaging (MRI) study that detects lesions suggestive for MS [3]. There is also the recognition of a prodromal stage of MS, that is only recently described. This may span a five to ten year period before neurologic onset of the disease, and can involve nonspecific fatigue, and gastrointestinal and bladder issues, among other symptoms [4]. RIS and the prodromal state are not yet recognized as distinct MS clinical phenotypes.

There are two major forms of MS. Relapsing disease involves clinical attacks, with stability in-between these episodes. This correlates with CNS focal inflammatory damage, and development of macroscopic MRI lesions. In contrast, progressive MS involves gradual clinical worsening, and represents neurodegenerative disease with microscopic injury. There are four defined clinical phenotypes [5]. About 85% to 90% of MS present with a clinically isolated syndrome (CIS), such as optic neuritis or transverse myelitis. This is a CNS inflammatory demyelinating syndrome that can be the first attack of relapsing MS (in perhaps 60% to 70% of cases). Some CIS patients will meet criteria for a definite diagnosis of MS, using the 2017 revised McDonald diagnostic criteria [6]. If they do not meet these criteria, they are categorized as CIS low risk or high risk for MS, based on whether the MRI shows silent lesions suggestive for MS. This is an important distinction, because CIS high risk will be offered MS disease modifying therapy (DMT). CIS low risk will be followed clinically and with serial MRIs, to document whether there is an ongoing damaging process. Of course, all patients should be worked up to exclude other causes of their neurologic picture.

Relapsing MS is clearly the major MS phenotype. However, 10% to 15% of MS begins with gradual worsening from onset, referred to as primary progressive MS (PPMS). The fourth and final MS clinical phenotype is secondary progressive MS (SPMS). It is believed that every relapsing patient has the possibility of transitioning to slow worsening SPMS, typically around mid-life (ages 45 to 55 years of age). The progressive MS phenotypes represent the clinical expression of CNS neurodegenerative injury to synapses, axons, and neurons. This appears to be the same process in both progressive phenotypes. Neurodegeneration is present at the earliest time point in the MS process, and continues over the disease lifetime. The mid-life association may be explained as crossing a threshold of loss, which makes clinical expression apparent.

The cause of MS is not clear, but three factors are recognized: genetics, environmental factors, and immune-mediated damage (Table 2). More than 233 genes have been associated with MS [7]. These are largely risk/susceptibility genes, with a few protection and disease severity genes. Gene associations may change based on racial/ethnic background. The strongest gene association (10.5% of genetic variance) is the HLA-DRB1*15:01 haplotype.

Both genetic predisposition and sufficient environmental exposures are required to develop MS. In a recent provocative analysis, less than 7.3% of the population was suggested to be genetically vulnerable to MS [7]. Heterogeneous genetic combinations are involved, with an idiosyncratic MS susceptibility.

Specific environmental exposures, perhaps in sequence, appear necessary. First, there may be an intrauterine or early postnatal factor. This is suggested by the discrepancy in recurrence rates between twin and non-twin siblings, that concordant half twins are twice as likely to share the mother vs. the father, and that there is an impact of birth month on MS risk [7]. The second environmental effect occurs at or around puberty, before age 15 years, based on migration data risk. Adolescent obesity may play a role here. Third, the clinical onset of MS typically occurs much later, supporting one or more additional environmental factors. Even with the proper genetic and environmental background, more than 50% of such individuals will not develop clinical MS. Some will have silent disease.

The final etiologic factor in MS is an immune-mediated attack on the CNS. No critical auto-antigen has ever been shown for MS, so immune-mediated damage seems more accurate than autoimmune damage. There is an “outside-in” process which involves peripheral immune cells penetrating the CNS to result in focal inflammation, tissue injury, macroscopic lesions on MRI, and clinical relapses. However, there is also an “inside-out” in situ component of microglial activation, astrogliosis, diffuse low grade CNS inflammation, neurodegeneration, and injury to the blood brain barrier, as well as oligodendrocytes, ion channels, and microscopic damage, that occurs even in normal appearing CNS tissue. This accumulates over time and likely contributes to progression.

MS is not yet curable, but there are currently more than 25 disease modifying therapies (DMTs) (including generics), that cover ten distinct mechanisms of action (Table 3). These DMTs reduce future relapses, neurologic disability, and new MRI lesions. They all manipulate the immune system, and can be divided into three main groups: injectables, orals, and monoclonals. A final DMT, mitoxantrone, is an IV anthracenedione chemotherapeutic no longer used in MS due to its cardiotoxicity, a treatment-related leukemia, and alternative safer options.

### 1.2. Males

MS is a female predominant disease. That being said, 25% of MS individuals are male. There are very interesting sex differences when you focus on men. Men are less likely to develop MS, but if they do, they are more likely to develop PPMS. PPMS is the only MS phenotype that is not female predominant; it shows a fairly equal sex ratio. PPMS also has a decade later age of onset than relapsing MS. Most PPMS patients present in mid-life with a slowly worsening myelopathy, with progressive weakness of legs and abnormal gait.

Just based on their sex, MS men have a worse prognosis than women. They show poorer recovery from MS relapses, higher rates of brain volume loss, more cognitive impairment, greater disability development, and higher rates of transitioning to SPMS [11,12]. In a global MSBase registry study of *n* = 15,826 MS patients from 25 countries, *n* = 14,753 had relapse-onset MS. Males worsened annually on EDSS significantly more compared to females (0.133 vs. 0.112, *p* < 0.01). Women had a decreased risk of developing SPMS (*p* = 0.001). In contrast, PPMS (*n* = 1373) did not show a sex-based difference in EDSS worsening [13].

Men may also present with somewhat different symptoms than women. Relapsing MS men are more likely to have motor problems, and less likely to have optic neuritis [11].

There are limited studies evaluating the role of MS men in pregnancy. DMT use does not appear to be a factor [14,15,16,17]. Current consensus would be that there is no fertility issue for men with MS. That being said, a study involving the Danish MS Registry and National In Vitro Fertilization (IVF) registry did report an association between male fertility and MS [18]. Of *n* = 24,011 men with infertility (47%) vs. *n* = 27,052 (53%) without an issue, oligospermia was found in 35%. The infertility group had a higher risk of prevalent MS (*n* = 49 vs. *n* = 36, OR 1.61), and incident MS in follow up (*n* = 29 vs. *n* = 27 MS; HR 1.28). This might indicate hypogonadism, a shared genetic link, or a joint immune-mediated component. In a Truven Health MarketScan claims database, analyzed from 2001 to 2008, infertile men were identified by diagnosis and prescription codes. There were *n* = 33,077 infertile men, compared to *n* = 77,693 vasectomized men, and *n* = 330,770 age-matched control men [19]. Immune disorders were found in <0.1%. Infertile men showed increased risk for autoimmune disorders (that included MS, rheumatoid arthritis, psoriasis, thyroiditis and Grave’s disease).

In another small study of *n* = 68 MS men and *n* = 48 matched controls, the endocrine profile, hypothalamic pituitary-testes (HPT) axis, and semen quality were studied. The MS men showed decreased luteinizing hormone (LH), follicular stimulating hormone (FSH), and testosterone (*p* = 0.01). Results of injection of gonadotropin releasing hormone (GnRH) analogue did not show the expected increase in FSH and LH levels in MS men. Total sperm counts, motility, and percent of normal morphology were lower in MS vs. controls. Progressive MS men were found to have higher and more severe HPT axis abnormalities than relapsing MS men. Although the numbers are small, the author concluded that MS men are more likely to have hypogonadotropic hypogonadism and fertility impairment. Clearly these are provocative studies that need to be pursued. However, current available data does not support a clear negative impact on fertility in MS males.

Testosterone has been linked to MS. In animal models, testosterone shows anti-inflammatory as well as neuroprotective properties [20]. Up to 40% of men with MS are reported to have lowered levels, which have correlated with worse physical and cognitive disability. Lower prenatal testosterone levels, as documented by an increased 2D to 4D ratio of the second to fourth fingers, was associated with MS risk [21]. Obesity and elevated body mass index (BMI) during puberty (adolescence) appear to increase risk for MS; in males this obesity is associated with lower testosterone levels.

Testosterone has been tested in a pilot MS trial in ten men, with benefits on cognitive performance as measured by the paced auditory serial addition test, brain volume loss, and lean body mass [22]. There was no treatment effect on contrast lesions, T2 lesion volume, or EDSS. Testosterone therapy can have cardiovascular risk, which might argue for developing a safer biosynthetic product.

### 1.3. Females

MS is a female predominant disease. The current sex ratio is 3:1, and this ratio has been increasing over time [23,24,25]. MS is increasing among women, but this is limited to relapsing MS. It is likely that environmental factors contribute to this finding. Some studies suggest late onset MS is increasing [24], and PPMS may be decreasing [26].

### 1.4. Fertility

The current data supports no significant impact on fertility in MS women. That being said, there are some intriguing studies in males as noted in the previous section. There are also a few intriguing studies involving women. Blood anti-Mullerian hormone (AMH) levels, and ovarian volume and antral follicle count, are considered measures of ovarian reserve and fertility. They have been studied in several small MS series. There is conflicting data indicating that relapsing MS women vs. controls, or relapsing MS with active disease, show abnormalities consistent with impaired fertility [27,28,29,30]. A recent study reported that lower AMH levels (as a marker for ovarian aging) were associated with greater clinical disability, and greater total gray matter and cortical gray matter volume loss [31].

### 1.5. Comorbidities

Increasing attention is being paid to comorbid conditions in MS, which can attack CNS reserve and contribute to accelerated CNS aging. Comorbidities differ based on sex. Vascular comorbidities, such as diabetes, hypertension, hyperlipidemia and ischemic heart disease, increase with age. They are higher in men than women, and have been associated with more rapid MS worsening [32,33]. In one study, at the time of diagnosis, hypertension was 16% higher in MS women and 48% higher in MS men vs. controls. MS women showed higher rates of chronic lung disease, while MS men showed higher rates of diabetes, epilepsy, depression and anxiety [33]. Mental health comorbidity has been associated with increased disability worsening [34].

### 1.6. Neuroimaging

There is sexual dimorphism in the human brain, and healthy controls show sex-based differences in brain neuroimaging [35]. Men have larger brains and a higher percentage of white matter [36]. Women show higher percentage of gray matter, correcting for intracranial volume effect. Sex-based differences in the size and shape of specific brain structures are documented.

It is somewhat controversial, based on limited study sizes, whether there are true sex-based MRI differences in MS. In a 2009 literature review, no differences were found on T2 or T1 lesion burden, or on brain atrophy, magnetization transfer ratio or diffusion tensor imaging [37]. It has been suggested that men show fewer contrast lesions, greater gray matter volume loss, increased T1 hypointense lesions, and increased spinal cord axon loss. Men are reported to show greater retinal nerve fiber layer thinning after optic neuritis [38]. Matched male vs. female MS subjects showed cognitive deficits, along with decreased functional connectivity and network efficiency. Male MS subjects showed greater white matter diffusion changes, and impact on cognition.

A prior study reported gray matter and central atrophy more advanced in male patients, while white matter atrophy was more advanced in MS females [39]. A more recent study reported men vs. matched women with MS showed greater regional (thalamic and cortical) GM atrophy [40]. In a small study of *n* = 34 relapsing MS subjects, with females and males matched for age and disease duration, diffusion tensor imaging was used to study optic radiation lesions and non-lesional normal-appearing white matter at baseline and 12 months. Axial diffusivity (which detects axonal loss) and radial diffusivity (which detects myelin loss) increased significantly over one year in males (2.7% and 4.6%) but not females (0.9% and 0.7%) [41]. This is an area that needs large scale studies, perhaps using machine learning techniques.

### 1.7. Inheritance

MS individuals may be concerned that they can pass on the disease to their children. This is an important counseling issue; there is no gene that passes on MS. Only 20% of MS individuals report a positive family history of MS, although if you were to probe relatives with MRIs and cerebrospinal fluid (CSF) examinations, you would detect a small minority with abnormalities suggestive for silent MS. MS is not considered an inherited disease. That being said, a close relative such as a sibling or parent with MS increases the risk from about 0.13% to the 2–3% range (with an affected parent the rate is 2% to 2.5%; with a sibling it is 2.7%) [42].

### 1.8. Pregnancy

Pregnancy is a major topic in MS, because the prototypic patient is a young woman of childbearing age. You do not like to use any unnecessary medication during pregnancy. As a rule of thumb, you need at least a thousand human pregnancy exposures to judge drug safety. Thousands of exposures exist for the earliest injectable DMTs, glatiramer acetate and the interferon betas, without any teratogenicity [43]. By expert consensus, they do not need a pregnancy washout, they are considered safe to use during pregnancy, and safe to use while breastfeeding [44,45]. Glatiramer acetate has been used in several hundred MS pregnancies, more so than the interferon betas. Unfortunately, none of the other DMTs have sufficient exposures to comment on, although none are proven to be human teratogens. It has been argued that the fumarates have no real documented animal teratogenicity, and have such a short half-life (one hour) that they probably do not warrant a pregnancy washout. In addition, natalizumab is associated with potentially severe rebound activity during pregnancy. In fact, no pregnancy washout is recommended for this monoclonal antibody, and it is often used through the first one to two trimesters of pregnancy. The oral S1P-receptor modulators also carry risk of rebound, but they are not used in pregnancy due to developmental toxicity in animal studies. The S1P receptor is involved in vascular and neural formation during embryogenesis [46]. Fingolimod takes six weeks to wash out. Siponimod and ponesimod are much shorter (one week), while ozanimod is longer (eight weeks). Oral teriflunomide is teratogenic in animal models, but to date has not been shown to be teratogenic in humans. It must be washed out over 11 days with an oral cholestyramine regimen that lowers drug blood level to undetectable (≤0.02 mcg/mL). Teriflunomide can enter semen. In a study of ten couples where the male was on teriflunomide, and as a group their mean blood level was 42.3 mcg/mL (10.07–142.84), blood levels were detected in four women at a mean of 0.045 (0.022–0.077) mcg/mL [47].

It has been argued that DMTs with infrequent dosing (oral cladribine, alemtuzumab, anti-CD20s) allow specific drug-free periods in which to try to get pregnant, and may offer attractive options.

MS individuals are counseled that their disease has little to no impact on ability to conceive, pregnancy, or fetal status. An MS pregnancy is not considered high risk, with no proven impact on fertility (see earlier discussions), and no increase in birth defects. There is no consistent increase in abortions, ectopic pregnancies, or assisted vaginal or cesarean deliveries. There is no limitation on delivery method or anesthetic, and no documented impact from a father with MS. Pregnancy does not worsen long-term MS prognosis. In fact, a few studies have suggested improved long-term prognosis [44]. Pregnancy seems to decrease risk of CIS. A recent report from the Italian Pregnancy Dataset used propensity matching to examine *n* = 230 pregnant MS women and *n* = 102 control MS women [48]. They reported that relapses in the year prior to conception increased the risk for long term disability over a 6.5 year follow up.

It is worth pointing out that the majority of pregnant MS women have relapsing MS (87% to 97%) without marked disability. There is not reliable data on pregnancy in progressive MS women with higher disability.

Pregnancy has an important impact on MS disease activity, at least regarding the relapse/MRI lesion components. MS disease activity decreases during pregnancy, particularly in the last trimester. This likely reflects ongoing immune changes, including rising hormone levels, that promote an immunotolerant state. However, relapse rate rebounds to increase postpartum, particularly in the first three months, before returning to pre-pregnancy levels. This pattern has been confirmed in a multitude of studies [49,50]. The one exception which did not find a postpartum increase in relapse rate was a Kaiser Permanente study [51]. However, a recent meta-analysis of pregnancy studies since 1998 looked at 7034 pregnancies in *n* = 6430 MS women [52]. Annualized relapse rate (ARR) was 0.57 pre-pregnancy. It was 0.36 in the first trimester, 0.29 in the second trimester, and 0.16 in the third trimester. The postpartum rebound rate was 0.85. In this meta-analysis, ARRs were decreasing over time, but the pattern remained intact. There was no documentable impact of diagnosis, DMT exposure, or breastfeeding. Studies using claims data were noted to have the lowest ARRs.

Relapses can occur during pregnancy [53]. They can be treated with short courses of steroids. Methylprednisolone should be used, not dexamethasone which crosses the placenta. Steroids can be used in the first trimester, since the most recent studies since 2008 do not confirm a cleft-lip or cleft palate risk [54,55]. MRIs can be done without risk at any time during pregnancy. However, gadolinium contrast is avoided (it passes the placenta) unless it provides critical information.

Of course, symptomatic drug therapy should be minimized during pregnancy. Vitamin D levels should be normalized, and appropriate folic acid and prenatal vitamins are used. There should be no smoking, limited to no alcohol use, and good sleep hygiene. All pregnant patients should have received the COVID-19 vaccine.

### 1.9. Breastfeeding

There has been a focus on breastfeeding, especially with regard to whether it precludes use of DMTs.

There is no evidence for a negative effect of breastfeeding. Most (but not all) studies report a decrease in MS disease activity with breastfeeding, but it appears that exclusive breastfeeding (less than one bottle daily) is necessary. Breastfeeding is associated with lactational amenorrhea. A recent systematic review and meta-analysis concluded that it was likely protective against postpartum relapses [56]. The degree of MS disease suppression is not clear; is it equivalent to a DMT? Current opinion is that you can breastfeed even though you are receiving steroids (a 4 h delay is optional), and that you can breastfeed while on treatment with glatiramer acetate, the interferon betas, and the monoclonal antibodies. IgG will enter breast milk, but should be partially destroyed in the infant’s GI tract. There are provocative studies that breastfeeding for a minimum of 15 months may lower maternal risk for future MS [57]. There is also a study indicating that breastfeeding may lower the infant’s risk for MS [58]. Another study found a lowered risk in male infants, with a synergistic effect of HLA-DRB1*15:01 [59].

### 1.10. Family Planning Issues

The American Academy of Neurology 2018 Practice Guidelines on the MS DMTs endorsed, when starting a therapy in women of childbearing age, that reproductive plans should be monitored, and that MS women should be counseled regarding reproductive risks and use of birth control during DMT use [60]. They also recommended stopping a DMT electively for planned pregnancies, unless MS activity risk outweighed the DMT risk. They advised to discontinue a DMT if a person became pregnant, and not to initiate a DMT during pregnancy, unless risk of activity during pregnancy outweighed the DMT risk.

### 1.11. Contraception

There is no data to suggest any contraceptive use is harmful to MS. Recent US Centers for Disease Control Guidance only noted that combined hormonal contraceptives might be of concern in women with MS who have prolonged immobility with risk of venous thromboembolism [61]. These contraceptives may also have potential negative interactions with drugs such as modafinil, carbamazepine, topiramate, and oxcarbazepine.

Among the various types of contraception, long acting reversible contraceptives (LARC) stand out. They involve intrauterine devices and hormonal implantable rods that last three to five years, or up to ten years. They are 99% or more effective, once implanted require no further compliance, and can be removed at any time. In a Denmark and North American study of 17,954 women (not MS), they examined the delay in discontinuing contraception and time to fecundability. LARC had the shortest delay (two cycles) [62]. This is another appealing feature to raise in patient discussions.

### 1.12. Assisted Reproductive Technology (ART)/IVF

About 10% to 20% of couples in western countries experience infertility. Up to 1% of live births are the result of ART/IVF, and its use in MS is reported in as high as 14% of pregnancies. A recent pooled analysis of six studies, encompassing 164 cycles, and a meta-analysis of all studies since 2017 (220 cycles), reported that the ARR increased in the three months after unsuccessful ART/IVF (*p* ≤ 0.01) [63]. This was true regardless of the protocol used (gonadotropin releasing hormone agonists or antagonists, or non-gonadotrophin releasing hormone). In a small sample, continuing DMT during the ART/IVF procedure prevented this activity increase.

### 1.13. Sexual Dysfunction

Sexual dysfunction is said to affect 40% to 80% of MS women, and 50% to 90% of MS men. It is an underdiagnosed and undertreated disease symptom that often worsens over time. It is associated with a poorer quality of life. It reflects sequelae of primary issues (damage to the CNS, autonomic dysfunction), secondary issues such as fatigue, depression, bladder/bowel or spasticity issues, and tertiary issues (psychosocial body image, culture, relational issues). The major problems are lack of sexual interest, decreased orgasm, decreased vaginal lubrication in women, and ejaculatory dysfunction in men. This is an important area to consider to assure symptomatic MS therapy is optimized.

### 1.14. Menopause

Menopause is defined as the permanent cessation of menstrual periods (for 12 consecutive months). The median age for women is 51 to 52 years. Menopause is a natural phenomenon, but can be iatrogenic due to surgery, chemotherapy, or radiation. Estrogen levels fall. Common menopausal symptoms are vasomotor issues (hot flashes, night sweats), sleep disruption, fatigue, depression, cognitive complaints, urinary changes, and sexual disruption. Menopause is accompanied by increased risk for cardiac disease and osteoporosis.

Menopausal hormonal therapy is offered for symptomatic relief of marked vasomotor issues or genitourinary issues. Osteoporosis prophylaxis may be offered. Other treatments involve herbal supplements (soy and black cohosh) and off-label use of SSRIs and anticonvulsants [64]. The hormonal therapy involves low estrogen (in an oral, patch, topical, or vaginal formulation). Combination therapy with synthetic progesterone is used for women who have a uterus (to avoid endometrial cancer).

Age at menopause is not different for MS, but it coincides with the age-related risk period for progressive disease. Menopause can certainly produce symptoms that overlap with MS. MS individuals have reported worsening of MS symptoms (often related to hot flashes), and greater disability at menopause. MS individuals treated with hormone replacement therapy have noted symptomatic improvement and stable disability.

Andropause has been used to describe male menopause. It is late onset age-related hypogonadism, with slowly decreasing testosterone levels of 1% to 1.4% annually. This starts from the mid-30s onward. Late onset hypogonadism requires signs of testosterone deficiency: low libido, erectile dysfunction, decreased muscle mass and strength, increased body fat, decreased bone mineral density and osteoporosis, and decreased vitality and depressed mood [65].

Menopause is an understudied area for both women and men with MS.

### 1.15. Menses

The menstrual cycle is controlled by the hypothalamic-pituitary-ovarian axis [66]. There are four phases: menstruation, follicular phase, ovulation, and luteal phase. It is known that estrogen and progesterone levels can modulate neural circuits, especially within the cortico-limbic regions [67]. Formal studies of the menstrual cycle in MS are limited. Rarely menstrual-related relapses are described. There may also be pseudo-relapses [68]. A very early study on *n* = 8 relapsing MS women reported a relationship of progesterone to estradiol ratio during the luteal phase on the number and volume of contrast brain lesions. In one analysis, MS patients vs. controls reported more irregular cycles, and more premenstrual symptoms [69]. MS women may have worsening of symptoms prior to menstruation. In a study of cognitive and physical performance, MS women showed worse premenstrual cognitive and physical function [70]. However, clearly, more studies are needed to understand menstrual cycle links to MS symptoms or disease activity.

## 2. Summary

Both the human CNS and immune systems show documented sex-based differences. Therefore, it is not surprising that MS involves many sex-based differences, and these could provide insight into the disease. Genetic, environmental, immunologic and hormonal factors likely contribute to these differences. Studies in this field directly led to trials evaluating sex hormones (estriol, testosterone) as treatments for women and men with MS. However, there are issues with using these hormones. Perhaps designer hormones, that possess beneficial CNS and immune effects, without unwanted endocrinologic effects, will play a future role. That being said, sex-based studies have led to important changes in the clinical care, practice, and counseling of MS individuals. Ongoing studies to understand the sex-based differences in MS frequency and severity holds the promise of novel insight into this major neuroinflammatory disease of young adults.

## Figures and Tables

**Table 1:** MS spectrum and **clinical phenotypes**.

● Population at risk
Asymptomatic
● Silent MS ● Radiologically isolated syndrome
Symptomatic
● Prodromal MS ● **Clinically isolated syndrome/relapsing MS**
○ 3:1 female ratio ○ 85% to 90% of MS at presentation
● **Primary progressive MS**
○ Equal sex ratio ○ 10% to 15% of MS at presentation
● **Secondary progressive MS**
○ Prior relapsing MS ○ Transition to progressive MS linked to midlife

**Table 2:** Etiology of MS.

Genetics
● >233 risk/susceptibility genes ● Some protection and disease severity genes
Environmental
● Birth month (northern hemispheres shows excess MS births in spring, and a decrease in autumn; southern hemisphere shows the reverse [8,9,10]) ● UV radiation/sunlight exposure ● EBV seropositivity; clinical illness (mononucleosis) ● Adolescent obesity ● Vitamin D deficiency (in white demographics) ● Tobacco smoking ● Permissive gut microbiome
Immunologic
● Focal inflammation with CNS damage
○ Edema, inflammation early, variable demyelination, axon injury, gliosis, loss of oligodendrocytes/neurons ○ Relapses ○ Contrast lesions, new/enlarging T2 lesions on neuroimaging
● Neurodegeneration
○ Synapse, axon, neuron damage ○ Microscopic injury on neuroimaging ○ CNS tissue volume loss

**Table 3:** MS DMTs.

Injectables
● Interferon betas (IFNβs)
○ IFNβ-1a 30 mcg IM weekly ○ IFNβ-1a 44 mcg (or 22 mcg) SC 3x weekly ○ Pegylated IFNβ-1a 125 mcg SC or IM Q14 days ○ IFNβ-1b 250 mcg SC QOD (brand and bioidentical)
● Glatiramer acetate
○ 20 mg SC daily (brand, generics) ○ 40 mg SC 3x weekly (brand, generics)
Orals
● Sphingosine-1P-receptor (S1P-R) modulators
○ Fingolimod 0.5 mg PO daily ○ Siponimod 2 mg PO daily ○ Ozanimod 1 mg (0.92 mg) PO daily ○ Ponesimod 20 mg PO daily
● Fumarates
○ Dimethyl fumarate 240 mg PO 2x daily (brand, generics) ○ Diroximel fumarate 462 mg PO 2x daily ○ Monomethyl fumarate 190 mg PO 2x daily
● Teriflunomide
○ 14 mg (or 7 mg) PO daily
● Cladribine
○ 3.5 mg/kg (8–10 treatment days in yr 1, during months 1 and 2; 8–10 treatment days in yr 2, during months 1 and 2)
Monoclonals
● Natalizumab
○ 300 mg IV Q4 weeks
● Alemtuzumab
○ 12 mg IV daily × 5 days in yr 1; 12 mg IV daily × 3 days in yr 2
● Anti-CD20s
○ Ocrelizumab 600 mg IV Q6 months ○ Ofatumumab 20 mg SC monthly (after 20 mg SC wks 0, 1, 2)
Chemotherapeutics
● Mitoxantrone
○ 12 mg/m^2^ IV Q3 months (lifetime maximum 140 mg/m^2^)

## Data Availability

Not pertinent.
